# Modulation of Attentional Bias to Drug and Affective Cues by Therapeutic and Neuropsychological Factors in Patients With Opioid Use Disorder on Methadone Maintenance Therapy

**DOI:** 10.3389/fpsyt.2021.780208

**Published:** 2022-01-12

**Authors:** Wenhui Li, Jin Huang, Nan Zhang, Kathrin Weidacker, Jun Li, Valerie Voon, Chuansheng Wang, Chencheng Zhang

**Affiliations:** ^1^Department of Psychiatry, The Second Affiliated Hospital of Xinxiang Medical University, Xinxiang, China; ^2^Shanghai Jiao Tong University School of Medicine, Shanghai, China; ^3^Research Center of Brain and Cognitive Neuroscience, Liaoning Normal University, Dalian, China; ^4^School of Psychology, Swansea University, Wales, United Kingdom; ^5^School of Information Science and Technology, ShanghaiTech University, Shanghai, China; ^6^Department of Psychiatry, University of Cambridge, Cambridge, United Kingdom; ^7^Department of Neurosurgery, Ruijin Hospital, Shanghai Jiao Tong University School of Medicine, Shanghai, China; ^8^Center for Functional Neurosurgery, Ruijin Hospital, Shanghai Jiao Tong University School of Medicine, Shanghai, China; ^9^Shanghai Research Center for Brain Science and Brain-Inspired Technology, Shanghai, China

**Keywords:** opioid addiction, methadone maintenance therapy, attentional bias, treatment duration, impulsivity, heroin

## Abstract

**Objective:** Abnormal selective attention to drug cues and negative affect is observed in patients with substance dependence, and it is closely associated with drug addiction and relapse. Methadone maintenance is an effective replacement therapy to treat heroin addiction, which significantly reduces the relapse rate. The present study examines whether the patients with opioid use disorder on chronic methadone maintenance therapy exhibit abnormal attentional bias to drug cues and negative-affective cues. Moreover, its relation to therapeutic and neuropsychological factors is also examined.

**Methods:** Seventy-nine patients with opioid use disorder under chronic methadone maintenance therapy and 73 age-, sex-, and education-matched healthy controls were recruited and assessed for attentional bias to drug cues and negative affect using a dot-probe detection task. Correlational analysis was used to examine the relationships between the attentional bias and the demographic, therapeutic, and neuropsychological factors.

**Results:** No significant overall patient-control group difference is observed in drug-related or negative-affective-related attentional bias scores. In the patient group, however, a significant negative correlation is found between the attentional bias scores to negative-affective cues and the duration of methadone treatment (*p* = 0.027), with the patients receiving longer methadone treatment showing less attentional avoidance to negative-affective cues. A significant positive correlation is found between the negative affect-induced bias and the impulsivity score (*p* = 0.006), with more impulsive patients showing higher attentional avoidance to negative affective cues than less impulsive patients. Additionally, the patients detect a smaller percentage of probe stimuli following the drug (*p* = 0.029) or negative-affective pictures (*p* = 0.009) than the healthy controls.

**Conclusion:** The results of the present study indicate that the patients under chronic methadone maintenance therapy show normalized attentional bias to drug and negative-affective cues, confirming the involuntary attention of the patients is not abnormally captured by external drug or negative-affective clues. Our findings also highlight that the attentional avoidance of negative-affective cues is modulated by the duration of methadone treatment and the impulsivity level in the patients.

## Introduction

The patients with drug use disorder allocate more attention to drug-related information in the environment, which may result in partial or full relapse back to addictive behavior (1). A number of studies have provided evidence supporting the hypothesized role of drug-related attentional bias (AB) in drug addiction. Drug-related attentional bias is associated with the severity of the addiction (2, 3), drug craving (4), and relapse to drug use after a period of abstinence (5). Successful abstinence may reduce the attentional bias in former drug-addicted patients (6). Attention control training may reduce attentional bias to drug cues and facilitate abstinence (7). Attentional bias to drug cues has been found in heroin addicts (3, 8), which may be mediated by enduring, perhaps permanent, changes within the brain's reward system (9).

Besides selective attention to drug-related cues, the seeking for relief in negative affect may also be related to drug use and relapse in drug-addicted patients (10). The patients with substance use disorder show a higher level of depression, anxiety, and impulsivity (11, 12). Those participants with substance abuse who have elevated negative affect report significantly higher ratings for cues of abused substances (13). Smokers show significant attentional biases to both smoking-related and negative-affect words (14). Acute induction of negative affect increases heroin seeking in the addicts, particularly in those who report greater subjective reactivity to negative triggers (15), suggesting addicts whose mood is more negatively influenced by external stimuli are at higher risk for drug use.

Methadone maintenance is an effective replacement therapy to treat heroin addiction, which significantly reduces the relapse rate (16). It reduces the negative feelings caused by withdrawal symptoms and cravings for opioids (17). Studies investigating whether patients with opioid use disorder on methadone maintenance therapy (MMT) show abnormal reactivity to drug cues and negative-affective cues are limited. A functional magnetic resonance study shows that heroin-addicted patients treated with MMT still exhibit enhanced brain responses to heroin-related visual stimuli, even just after their regular daily methadone dose (18). On the other hand, a review of the existing literature on attentional bias to drug addiction indicates less clear-cut and inconsistent results (19). For example, several studies using behavioral measures of selective attention reveal no drug-related attentional bias among opioid-addicted patients who receive MMT (20, 21). The inconsistent findings reported in the literature could result from methodological differences between the studies, or from differences in patient samples examined (19). Notably, the sample sizes of these studies are relatively small (around 20 participants in each study), and the duration of MMT is relatively short or not specified. There is also a lack of investigation on the relationship between attentional bias and demographic, therapeutic, and neuropsychological factors in the patients, for instance, anxiety and impulsivity, which are known to be associated with substance use and addiction (22–25).

In the present study, we employ a dot-probe detection task to assess drug-related and negative-affective-related attentional bias in opioid-addicted patients under chronic MMT, compared with matched healthy control participants (HCs). Moreover, the relationship between attentional bias and demographic, therapeutic, and neuropsychological factors is also examined. We hypothesized that opioid-addicted patients under chronic MMT might show normalized or mildly abnormal attentional bias to drug cues and negative-affective cues, based on the fact that methadone effectively treats heroin addiction and significantly reduce the relapse of heroin use. We also hypothesized that the attentional bias in the patients might be modulated by certain therapeutic or neuropsychological factors such as daily methadone dosage, duration of MMT, depression, anxiety, or impulsivity level.

## Materials and Methods

### Study Participants

From June 2018 to January 2019, we recruited 79 opioid-addicted patients from three MMT clinics located in Shanghai, along with 73 age-, sex-, and education-matched HCs who were recruited from the community through advertisements and assessed attentional bias to drug cues and negative affect using a dot-probe detection task. All patients were diagnosed with opioid use disorder by DSM-IV (26, 27). Patients included were treated with methadone for at least 1 month before the study and were not currently using any addictive drugs other than methadone, as verified by a urine test. The exclusion criteria for both participant groups include the presence of severe physical disease or mental disorders, such as psychotic, anxiety, and mood disorders. The procedures of this study were approved by the Ethics Committees of Ruijin Hospital, Shanghai Jiao Tong University School of Medicine. Informed consent was obtained from all participants after the study was fully explained. The participants received monetary compensation for their participation.

### Demographic, Neuropsychological, and Therapeutic Data Collection

We collected demographic information (age, sex, and years of education) from each participant. Additionally, we collected data of: (a) tobacco smoking status and nicotine dependency, assessed by the Fagerström Test for Nicotine Dependence (FTND) (28); (b) alcohol use status and dependency, using the Alcohol Use Disorders Identification Test (AUDIT) (29); (c) depressive symptoms, assessed by the Beck Depression Inventory (BDI) (30); (d) state and trait anxiety, measured by the State-Trait Anxiety Inventory (STAI) (31); and (e) impulsivity, evaluated by the UPPS-P, which is a 59-item self-report scale purported to measure five distinct aspects or dimensions of impulsivity, namely positive urgency, negative urgency, sensation seeking, lack of premeditation, and lack of perseverance (32).

The BDI has a high internal consistency with a coefficient alpha of 0.93, and the total score of 0-13 is considered minimal depression, 14-19 is mild, 20-28 is moderate, and 29-63 is severe (30). The STAI has a coefficient alpha of 0.86, and the range for each subset (STAI-S or STAI-T) is 20-80, the higher score indicating greater anxiety, with a cut point of 40 suggested to detect clinically significant symptoms for STAI-S (33). Positive Urgency and Negative Urgency subscales measure the tendencies to act rashly under extreme positive and negative emotions; Sensation Seeking subscale measures the tendency to seek novel and thrilling experiences; Lack of Premeditation subscale measures the tendency to act without thinking; and Lack of Perseverance subscale measures the inability to remain focused on a task (32).

The therapeutic data were collected from the patients, including the current daily dosage of methadone, the duration of MMT, and the subjective rating of opiate withdrawal effects, measured by the Subjective Opiate Withdrawal Scale (SOWS), with the total scores for possible range from 0 to 64 (34).

### Dot Probe Detection Task

**Figure 1:**
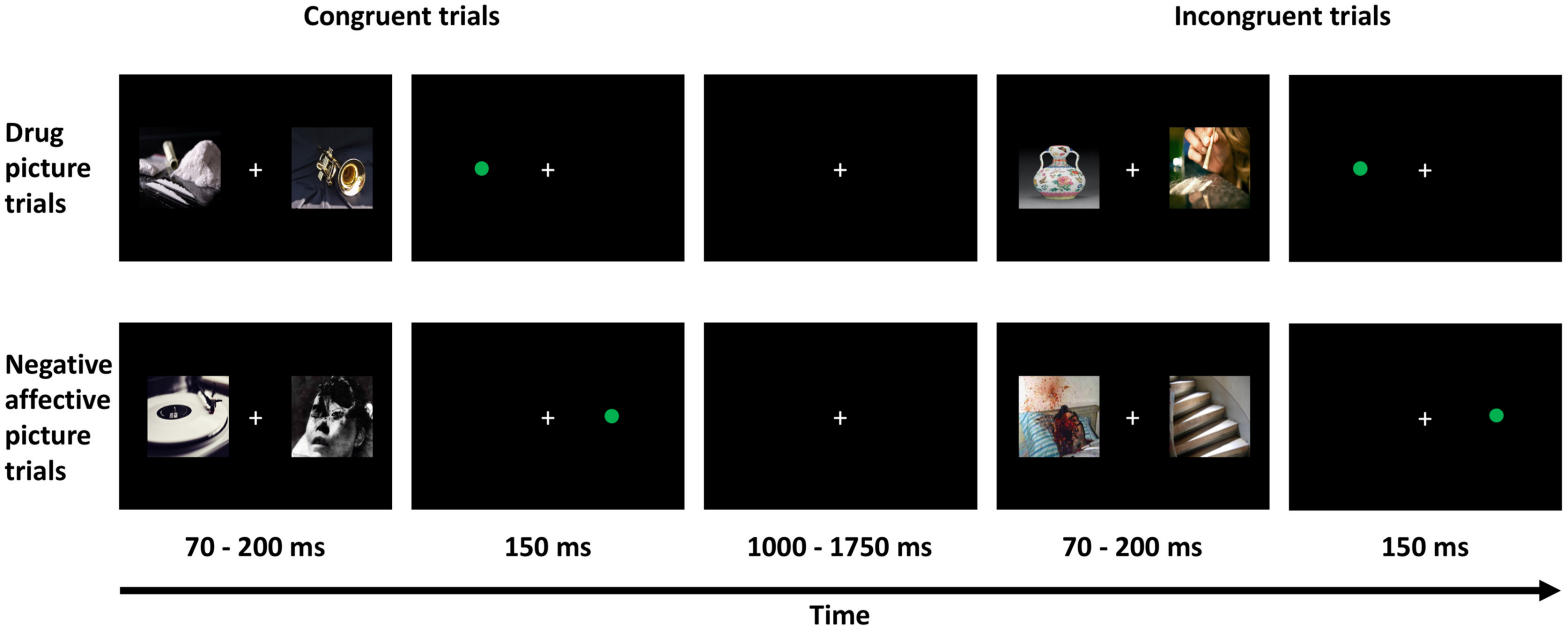
The paradigm of dot-probe detection task.

A visual dot-probe detection task was utilized to assess attentional bias to drug-related pictures as well as an attentional bias to emotionally negative pictures. The participants were sitting in front of a 14-inch computer screen, with a distance of 30 cm from eyes to the screen, while performing the task. There are a total of 96 trials in this task. The task paradigm is presented in Figure 1. For each trial, two pictures were presented simultaneously and randomly on either the left or right side of the screen and lasted for a random duration of 70-200 ms. The picture pairs consisted of a drug-related picture (e.g., picture of drugs or drug paraphernalia) paired with a neutral picture (e.g., picture of an emotionally neutral object such as a household item or music instrument), or an emotionally negative picture (e.g., a picture of a severely injured person) paired with an emotionally neutral picture. Immediately following the offset of the stimulus pair, a probe stimulus (a green dot) was presented at one of the two previous stimulus locations for 150 ms. The probe was presented at either the same location (namely congruent trials) or the opposite location (namely incongruent trials) of the relevant target stimulus (i.e., the drug-related or emotionally negative picture). A fixation was presented among the trials with a random duration ranging from 1,000 to 1,750 ms. The participants were asked to indicate as quickly as possible the location of the probe, pressing either the “Z” key with the left-hand index finger if the probe appeared on the left side, or the “M” key with the right-hand index finger if the probe appeared on the right side of the screen. For each participant, an AB score to the drug-related pictures and an AB score to the emotionally negative pictures were obtained by subtracting the response time (RT) to probes on congruent trials from the RT to probes on incongruent trials. Additionally, the proportion of correctly detected probe stimuli on congruent and incongruent trials were also compared.

### Assessment of Executive Functions

Since attentional bias and neuropsychological scores may coincide with neurocognitive deficits, the executive functions of the participants were also assessed by three tasks on a 9.7-inch iPad (4th generation) using the Cambridge Neuropsychological Test Automated Battery (CANTAB) Connect Research iPad version (https://www.cambridgecognition.com/cantab): (a) Spatial Working Memory (SWM) measuring the function of working memory, with the number of errors made by the participants as the measure; (b) Paired Associative Learning (PAL) measuring the function of learning and memory, with the number of patterns reached and the number of errors made by the participants as the measures; and (c) Stockings of Cambridge (SOC) measuring the function of strategic planning, with the number of moves in 5-move trials and the number of trials solved in minimum moves as the measures. The paradigms of the tasks are described in detail in our previously published article (35).

### Statistical Analysis

Non-parametric analysis was used for between-group comparison and investigation of correlational relationships in the present study. We assessed patient-control group differences in demographic and neuropsychological characteristics using Mann-Whitney tests, including age, years of education, scores on depression, anxiety, and impulsivity scales, as well as the performance on executive functions. Chi-square tests were used to evaluate the group difference in sex, the proportion of smokers and alcohol drinkers. Moreover, we performed correlational analysis using Spearman's correlation coefficient to assess the direction and strength of the relationships between the AB scores and the demographic, therapeutic, and neuropsychological data within and across the groups. All statistical analyses were performed using SPSS version 22 (IBM Corp., Armonk, NY). *P* = 0.05 was set as the level of statistical significance. The figures were plotted using Prism GraphPad (version 8).

## Results

### Demographic, Therapeutic, and Neuropsychological Data

Eighty-three patients and eighty HCs participated in this study, and the data of four patients and seven HCs were removed from further statistical analysis due to not high enough accuracy achieved in the task (see section Patient-Control Differences in Performance on Dot Probe Detection Task). Table 1 summarizes the demographic data from the remaining 79 patients under MMT and 73 HCs, along with the data on substance use (tobacco smoking, alcohol use), depression, anxiety, impulsivity, and executive functions. For the patients, the current daily dosage of methadone, the duration of MMT, and the self-rated level of withdrawal symptoms.

**Table 1:** Demographic information and data on substance use, anxiety, depression, and impulsivity for patients under MMT and HC participants.

	**MMT group (*****n*** **=** **79)**		**HC group (*****n*** **=** **73)**		**Between-group comparison**	
	**M (SD)**	**Range**	**M (SD)**	**Range**	**Statistic**	***P*-value**
Age (years)	49.6 (8.2)	32-64	49.0 (7.4)	31-64	U = 2,740	0.598
Sex (M/F)	54/25		49/24		χ^2^(1) = 0.026	0.871
Education (years)	10.2 (1.9)	6-16	10.0 (2.5)	6-16	U = 2,430	0.085
Tobacco use (smokers/non-smokers)	76/3		29/44		χ^2^(1) = 56.655	**<0.001**
FTND score	4.7 (2.5)	0-10	3.4 (1.9)	1-8	U = 676.5	**0.015**
Alcohol use (drinkers/non-drinkers)	41/38		27/46		χ^2^(1) =3.413	0.065
AUDIT score	5.4 (7.0)	1-36	6.0 (6.5)	0-31	U = 486.5	0.284
MMT duration (months)	93.5 (53.4)	2-224				
Current methadone dosage (mg/day)	48.6 (30.2)	2-150				
SOWS score	8.5 (9.6)	0-39				
BDI score	14.8 (10.7)	0-38	7.6 (7.7)	0-44	U = 1,672	**<0.001**
STAI-S score	36.1 (11.5)	20-68	29.5 (7.8)	20-49	U = 1,909	**<0.001**
STAI-T score	42.4 (11.5)	23-71	34.8 (8.8)	21-55	U = 1,779	**<0.001**
UPPS-P1 score: positive urgency	30.0 (10.1)	11-56	24.5 (7.4)	14-46	U = 1,932	**<0.001**
UPPS-P2 score: negative urgency	28.5 (7.5)	13-45	24.2 (7.0)	12-43	U = 1,963	**<0.001**
UPPS-P3 score: sensation seeking	27.9 (7.4)	12-48	25.3 (6.9)	14-42	U = 2,284	**0.027**
UPPS-P4 score: lack of premeditation	21.8 (5.4)	11-36	20.5 (4.3)	11-33	U = 2,495	0.152
UPPS-P5 score: lack of perseverance	20.0 (4.1)	10-28	17.9 (4.1)	10-30	U = 2,023	**0.001**
SWM: number of errors	16.9 (8.4)	0-48	15.0 (9.8)	0-39	U = 2,551	0.221
PAL: number of patterns reached	7.2 (1.1)	4-8	7.5 (1.0)	4-8	U = 2,432	**0.039**
PAL: number of errors	28.5 (15.3)	4-59	23.6 (14.0)	1-59	U = 2,361	0.054
SOC: number of moves (five-move trials)	8.4 (2.3)	5-12	7.6 (2.2)	0-12	U = 2,368	0.057
SOC: Trials solved in minimum moves	6.2 (2.5)	1-11	7.2 (2.4)	2-12	U = 2,243	**0.017**

*A significant difference (p < 0.05) is in bold. AUDIT, Alcohol Use Disorders Identification Test; BDI, Beck Depression Inventory; FTND, Fagerström Test for Nicotine Dependence; HC, healthy control; MMT, methadone maintenance therapy; PAL, Paired Associative Learning; SOC, Stockings of Cambridge; SOWS, Subjective Opiate Withdrawal Scale; STAI-S, State-Trait Anxiety Inventory-state; STAI-T, State-Trait Anxiety Inventory-trait; SWM, Spatial Working Memory*.

No significant group differences are observed in the age, percentage of males, or years of education. The percentage of tobacco smokers is significantly higher in the patient group (96.2%) than in the HC group (39.7%), and the level of dependence on nicotine, as indexed by the FTND, is also significantly higher in the patients than in the HCs. The percentage of alcohol users and AUDIT scores are not significantly different in the patient group.

Furthermore, the patients display significantly higher levels of depression and anxiety, as reflected by the higher scores on the BDI and STAI, when compared with the scores in the HCs. Furthermore, the patients show significantly higher scores on the Positive Urgency, Negative Urgency, Sensation Seeking, and Lack of Perseverance subscales of the UPPS-P. The scores on the Lack of Premeditation subscale do not reach a significance level for between-group comparison.

The patients reach somewhat fewer patterns in the Paired Associative Learning task (*p* = 0.039), and achieve fewer trials in minimum moves in the Stockings of Cambridge task (*p* = 0.017), compared with HCs. No significant difference is observed in the performance on the Spatial Working Memory task (*p* = 0.221).

### Patient-Control Differences in Performance on Dot Probe Detection Task

After removing the participants who did not achieve a high enough accuracy (whose accuracy rate for congruent or incongruent trials was below 75%), the performance data from 79 patients and 73 HCs were submitted to further statistical analysis.

The performance on the dot-probe detection task for each group and the results of the statistical analysis are summarized in Table 2. The detection rate of the patient group is significantly lower than that of HCs when probe stimuli are preceded by pictures that contained drug-related content in the incongruent stimulus trials (*p* = 0.029). In addition, the detection rate of the patient group is significantly lower than that of HCs when probe stimuli are preceded by pictures that contain negative emotional content in the congruent stimulus trials (*p* = 0.009).

**Table 2:** Performance of dot-probe task for patients under MMT and HC participants.

**Performance**	**Stimulus type**		**MMT group (*****n*** **=** **79)**		**HC group (*****n*** **=** **73)**		**Between-group comparison**	
			**M (SD)**	**Range**	**M (SD)**	**Range**	**Statistic**	***P*-value**
Target detection rate (%)	Drug	C	95.3 (6.7)	66.7-100.0	97.1 (4.5)	80.0-100.0	U = 2,476	0.100
		IC	93.4 (7.8)	64.0-100.0	96.1 (5.1)	80.0-100.0	U = 2,315	**0.029**
	Negative affective	C	94.1 (7.2)	69.2-100.0	97.0 (4.5)	76.9-100.0	U = 2,222	**0.009**
		IC	94.9 (7.2)	63.0-100.0	97.1 (4.7)	69.2-100.0	U = 2,423	0.066
Response time (msec)	Drug	C	358.1 (56.9)	262.0-556.5	369.4 (57.5)	267.6-595.8	U = 2,569	0.248
		IC	362.2 (53.2)	266.6-543.4	372.4 (53.7)	269.7-587.6	U = 2,548	0.217
	Negative affective	C	359.7 (60.7)	641.3-259.5	371.4 (56.4)	268.2-593.7	U = 2,496	0.154
		IC	357.5 (51.5)	540.8-249.2	369.3 (50.5)	264.5-588.2	U = 2,480	0.138
AB score (msec)	Drug		−4.1 (20.3)	−53.5-35.8	−3.0 (24.0)	−85.2-69.8	U = 2,842	0.880
	Negative affective		2.2 (24.5)	−47.3-122.4	2.1 (23.4)	−51.9-68.5	U = 2,841	0.877

*A significant difference (p < 0.05) is in bold. AB, attentional bias; C, congruent; HC, healthy control; IC, incongruent; M, mean; MMT, methadone maintenance therapy; SD, standard deviation*.

**Figure 2:**
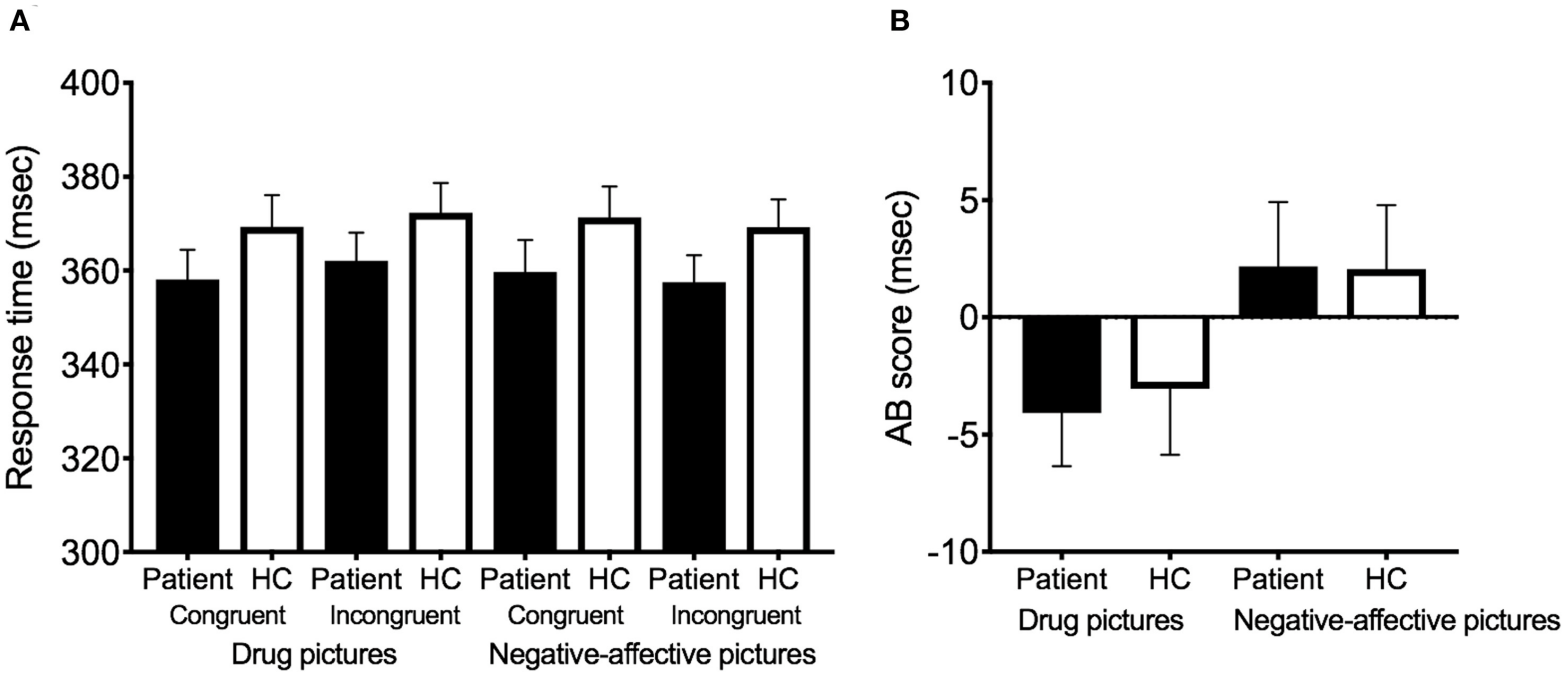
Response time and AB score in the patients (*n* = 79) and healthy controls (*n* = 73). **(A)** Response time for each condition. **(B)** AB score for each condition. The performance of the patients is indicated by black bars, and the performance of the healthy controls is indicated by white bars. Error bars indicate standard errors of the mean. AB, attentional bias; HC, healthy control.

No significant patient-control group differences are found in the RT to probe stimuli that follow the drug-related pictures or negative-affective pictures on either congruent or incongruent trials (*p* > 0.05; Figure 2A). There are no significant group differences in the drug-related or negative-affect-related AB scores (Figure 2B).

### Correlation Between AB Scores and Therapeutic or Neuropsychological Factors

Table 3 presents the results of the correlation analysis assessing the relationships between the AB scores and demographic information, scores on the therapeutic and neuropsychological scales, and executive functions within each group or in the combined patient-control sample.

**Table 3:** Bivariate correlation (Spearman correlation) between AB scores and demographics, substance use, scores on psychological measurements, and executive functions, for patients under MMT (*n* = 79) and HC participants (*n* = 73).

	**Variable**	**MMT group (*n* = 79)**	**HC group (*n* = 73)**	**All participants (pooled sample)**
AB score (drug)	Age	*r* = 0.112, *p =* 0.325	*r* = −0.046, *p =* 0.697	*r* = 0.042, *p =* 0.604
	Education	*r* = −0.048, *p =* 0.673	*r* = −0.095, *p =* 0.424	*r* = −0.059, *p =* 0.472
	FTND score	*r* = 0.044, *p =* 0.706	*r* = 0.234, *p =* 0.250	*r* = 0.058, *p =* 0.565
	AUDIT score	*r* = 0.025, *p =* 0.878	*r* = 0.016, *p =* 0.937	*r* = 0.057, *p =* 0.642
	MMT duration	*r* = −0.061, *p =* 0.595		
	Current methadone dosage	*r* = −0.113, *p =* 0.320		
	SOWS score	*r* = 0.127, *p =* 0.266		
	BDI score	*r* = −0.072, *p =* 0.531	*r* = 0.043, *p =* 0.718	*r* = −0.034, *p =* 0.674
	STAI-S score	*r* = −0.117, *p =* 0.305	*r* = 0.070, *p =* 0.556	*r* = −0.038, *p =* 0.641
	STAI-T score	*r* = −0.110, *p =* 0.335	*r* = 0.083, *p =* 0.483	*r* = −0.028, *p =* 0.729
	UPPS-P1 score: positive urgency	*r* = 0.003, *p =* 0.980	*r* = 0.047, *p =* 0.696	*r* = 0.017, *p =* 0.834
	UPPS-P2 score: negative urgency	*r* = 0.037, *p =* 0.744	*r* = 063, *p =* 0.597	*r* = 0.045, *p =* 0.586
	UPPS-P3 score: sensation seeking	*r* = 0.007, *p =* 0.953	*r* = 0.064, *p =* 0.591	*r* = 0.047, *p =* 0.565
	UPPS-P4 score: lack of premeditation	*r* = −0.052, *p =* 0.646	*r* = 0.173, *p =* 0.144	*r* = 0.052, *p =* 0.527
	UPPS-P5 score: lack of perseverance	*r* = 0.018, *p =* 0.873	*r* = 0.104, *p =* 0.382	*r* = 0.051, *p =* 0.536
	SWM: number of errors	*r* = −0.097, *p =* 0.398	*r* = −0.146, *p =* 0.218	*r* = −0.119, *p =* 0.145
	PAL: number of patterns reached	*r* = 0.056, *p =* 0.624	***r*** **=** **0.278**, ***p** **=*** **0.017**	*r* = 0.151, *p =* 0.064
	PAL: number of errors	*r* = 0.068, *p =* 0.550	*r* = −0.209, *p =* 0.076	*r* = −0.071, *p =* 0.385
	SOC: number of moves (five-move trials)	*r* = 0.023, *p =* 0.838	*r* = −0.012, *p =* 0.923	*r* = 0.012, *p =* 0.883
	SOC: Trials solved in minimum moves	*r* = −0.077, *p =* 0.498	*r* = 0.027, *p =* 0.818	*r* = −0.031, *p =* 0.709
AB score (negative)	Age	*r* = −0.054, *p =* 0.635	***r*** **=** **0.282**, ***p** **=*** **0.016**	*r* = 0.117, *p =* 0.152
	Education	*r* = −0.010, *p =* 0.381	*r* = −0.090, *p =* 0.451	*r* = −0.102, *p =* 0.210
	FTND score	*r* = 0.083, *p =* 0.474	***r*** **=** **0.398**, ***p** **=*** **0.044**	*r* = 0.104, *p =* 0.298
	AUDIT score	*r* = 0.129, *p =* 0.422	*r* = 0.104, *p =* 0.600	*r* = 0.185, *p =* 0.128
	MMT duration	***r*** **=** **−0.249**, ***p** **=*** **0.027**		
	Current methadone dosage	*r* = −0.142, *p =* 0.213		
	SOWS score	*r* = 0.122, *p =* 0.283		
	BDI score	*r* = −0.031, *p =* 0.784	*r* = 0.024, *p =* 0.839	*r* = −0.001, *p =* 0.988
	STAI-S score	*r* = 0.039, *p =* 0.735	*r* = −0.160, *p =* 0.176	*r* = −0.038, *p =* 0.646
	STAI-T score	*r* = 0.110, *p =* 0.335	*r* = −0.150, *p =* 0.207	*r* = 0.016, *p =* 0.842
	UPPS-P1 score: positive urgency	*r* = 0.077, *p =* 0.499	*r* = −0.020, *p =* 0.864	*r* = 0.047, *p = 0*.568
	UPPS-P2 score: negative urgency	*r* = 0.158, *p =* 0.165	*r* = −0.088, *p =* 0.460	*r* = 0.047, *p =* 0.565
	UPPS-P3 score: sensation seeking	*r* = −0.061, *p =* 0.591	*r* = 0.026, *p =* 0.828	*r* = 0.005, *p =* 0.952
	UPPS-P4 score: lack of premeditation	***r*** **=** **0.307**, ***p** **=*** **0.006**	*r* = −0.138, *p =* 0.243	*r* = 0.108, *p =* 0.185
	UPPS-P5 score: lack of perseverance	*r* = 0.221, *p =* 0.050	*r* = −0.069, *p =* 0.563	*r* = 0.082, *p =* 0.318
	SWM: number of errors	*r* = 0.209, *p =* 0.065	*r* = −0.006, *p =* 0.961	*r* = 0.087, *p =* 0.285
	PAL: number of patterns reached	*r* = −0.039, *p =* 0.731	*r* = 0.007, *p =* 0.950	*r* = −0.032, *p =* 0.670
	PAL: number of errors	*r* = −0.073, *p =* 0.524	*r* = 0.066, *p =* 0.578	*r* = 0.006, *p =* 0.943
	SOC: number of moves (five-move trials)	*r* = 0.033, *p =* 0.770	*r* = 0.024, *p =* 0.838	*r* = 0.041, *p =* 0.617
	SOC: Trials solved in minimum moves	*r* = 0.005, *p =* 0.964	*r* = 0.146, *p =* 0.217	*r* = 0.060, *p =* 0.466

*A significant correlation (p < 0.05) is in bold. AUDIT, Alcohol Use Disorders Identification Test; BDI, Beck Depression Inventory; FTND, Fagerström Test for Nicotine Dependence; HC, healthy control; MMT, methadone maintenance therapy; PAL, Paired Associative Learning; SOC, Stockings of Cambridge; SOWS, Subjective Opiate Withdrawal Scale; STAI-S, State-Trait Anxiety Inventory-state; STAI-T, State-Trait Anxiety Inventory-trait; SWM, Spatial Working Memory*.

**Figure 3:**
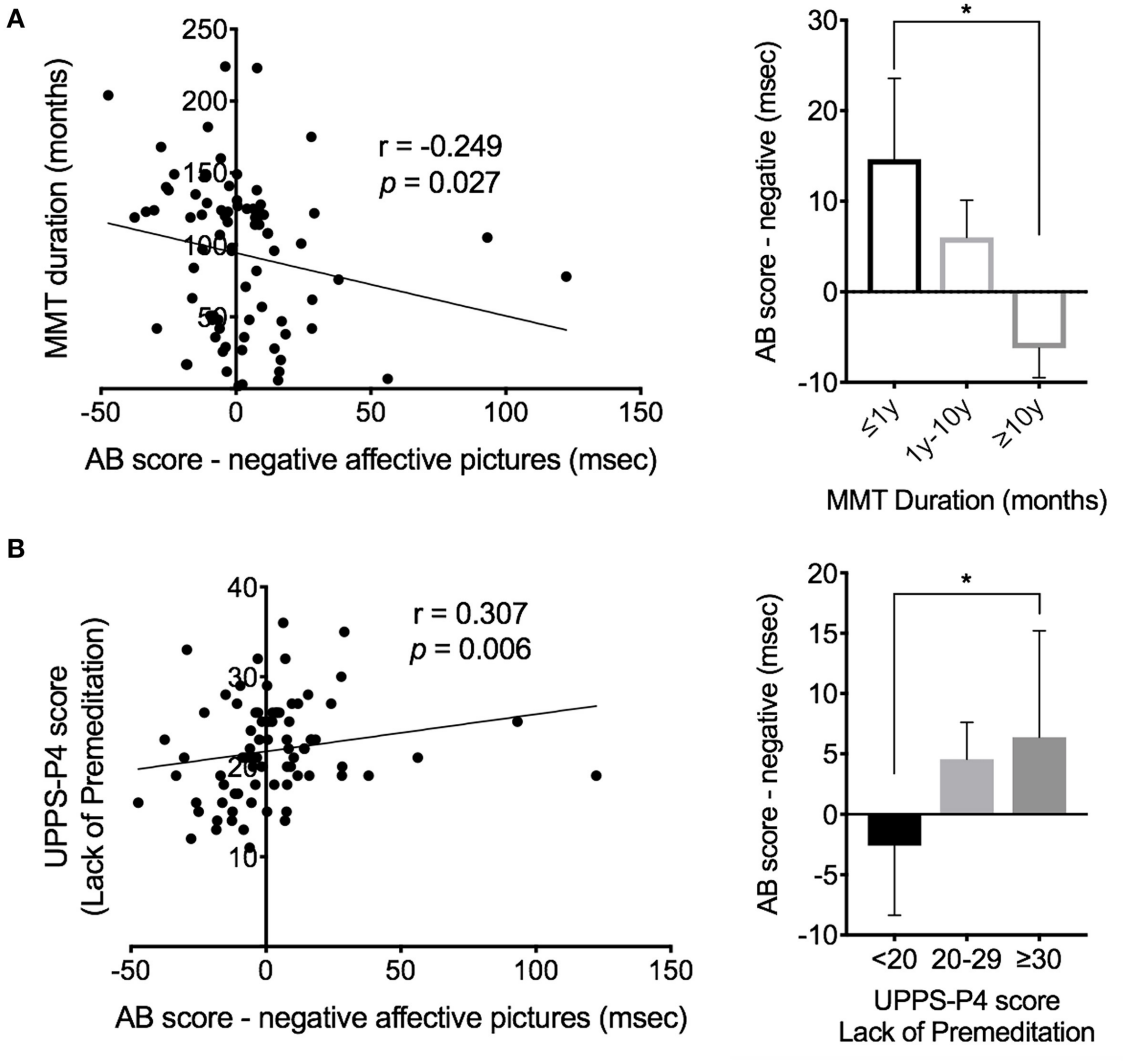
Modulation of MMT duration and trait of impulsivity on AB score of the patients (*n* = 79). **(A)** Correlation between the AB score and the MMT duration in the patients. **(B)** Correlation between the AB score and the UPPS-P4 score (score of Lack of Premeditation subscale). Error bars indicate standard errors of the mean. AB, attentional bias; MMT, methadone maintenance therapy; y, year. *Indicates a significance level of *p* < 0.05 by *post-hoc* testing using the Mann-Whitney test.

No significant correlation is found between the drug-related AB scores and the demographic, therapeutic, neuropsychological scores, or the performance on executive functions in the patient group. A significant negative correlation is observed between the negative-affect-induced AB scores and the duration of MMT (*p* = 0.027; Figure 3A). Additionally, a significant positive correlation is found between the negative-affect-induced AB scores and the subscale score on Lack of Premeditation in the patient group (*p* = 0.006; Figure 3B). We also performed the correlational analysis using the regression residual (incongruent RT as predictor regressed onto congruent RT as dependent variable), which is considered more appropriate than the difference in RT for bivariate correlations, and obtained similar statistical results with the use of AB scores (Table 4). This correlational relationship is also confirmed by Kruskal-Wallis analysis after dividing patients into subgroups by the MMT duration or the level of impulsivity on the Lack of Premeditation subscore, indicating the patients receiving longer methadone treatment show less attentional avoidance to negative-affective cues (Figure 3A; Table 5); and patients with higher impulsivity show higher attentional avoidance to negative affective cues than less impulsive patients (Figure 3B; Table 5).

**Table 4:** Bivariate correlation (Spearman correlation) between regression residuals and demographics, substance use, scores on psychological measurements, and executive functions, for patients under MMT (*n* = 79) and HC participants (*n* = 73).

	**Variable**	**MMT group (*n* = 79)**	**HC group (*n* = 73)**	**All participants (pooled sample)**
Regression residual (drug)	Age	*r* = 0.112, *p =* 0.325	*r* = −0.051, *p =* 0.671	*r* = 0.041, *p =* 0.619
	Education	*r* = −0.048, *p =* 0.673	*r* = −0.094, *p =* 0.427	*r* = −0.055, *p =* 0.497
	FTND score	*r* = 0.044, *p =* 0.706	*r* = 0.250, *p =* 0.218	*r* = 0.064, *p =* 0.525
	AUDIT score	*r* = 0.025, *p =* 0.878	*r* = 0.024, *p =* 0.904	*r* = 0.048, *p =* 0.694
	MMT duration	*r* = −0.061, *p =* 0.595		
	Current methadone dosage	*r* = −0.113, *p =* 0.320		
	SOWS score	*r* = 0.127, *p =* 0.266		
	BDI score	*r* = −0.072, *p =* 0.531	*r* = 0.038, *p =* 0.751	*r* = −0.027, *p =* 0.743
	STAI-S score	*r* = −0.117, *p =* 0.305	*r* = 0.063, *p =* 0.596	*r* = −0.035, *p =* 0.666
	STAI-T score	*r* = −0.110, *p =* 0.335	*r* = 0.086, *p =* 0.470	*r* = −0.021, *p =* 0.794
	UPPS-P1 score: positive urgency	*r* = 0.003, *p =* 0.980	*r* = 0.051, *p =* 0.666	*r* = 0.026, *p =* 0.747
	UPPS-P2 score: negative urgency	*r* = 0.037, *p =* 0.744	*r* = 0.069, *p =* 0.561	*r* = 0.052, *p =* 0.521
	UPPS-P3 score: sensation seeking	*r* = 0.007, *p =* 0.953	*r* = 0.070, *p =* 0.559	*r* = 0.058, *p =* 0.482
	UPPS-P4 score: lack of premeditation	*r* = −0.052, *p =* 0.646	*r* = 0.181, *p =* 0.125	*r* = 0.054, *p =* 0.507
	UPPS-P5 score: lack of perseverance	*r* = 0.018, *p =* 0.873	*r* = 0.121, *p =* 0.309	*r* = 0.063, *p =* 0.439
	SWM: number of errors	*r* = −0.097, *p =* 0.398	*r* = −0.135, *p =* 0.256	*r* = −0.112, *p =* 0.170
	PAL: number of patterns reached	*r* = 0.056, *p =* 0.624	***r*** **=** **0.274**, ***p** **=*** **0.019**	*r* = 0.144, *p =* 0.077
	PAL: number of errors	*r* = 0.068, *p =* 0.550	*r* = −0.209, *p =* 0.075	*r* = −0.070, *p =* 0.394
	SOC: number of moves (5-move trials)	*r* = 0.023, *p =* 0.838	*r* = −0.017, *p =* 0.886	*r* = 0.009, *p =* 0.912
	SOC: Trials solved in minimum moves	*r* = −0.077, *p =* 0.498	*r* = 0.023, *p =* 0.844	*r* = −0.036, *p =* 0.659
Regression residual (negative)	Age	*r* = −0.122, *p =* 0.285	***r*** **=** **0.282**, ***p** **=*** **0.016**	*r* = 0.078, *p =* 0337
	Education	*r* = −0.125, *p =* 0.274	*r* = −0.085, *p =* 0.473	*r* = −0.111, *p =* 0.174
	FTND score	*r* = 0.088, *p =* 0.447	***r*** **=** **0.406**, ***p** **=*** **0.040**	*r* = 0.109, *p =* 0.274
	AUDIT score	*r* = 0.117, *p =* 0.466	*r* = 0.124, *p =* 0.530	*r* = 0.177, *p =* 0.146
	MMT duration	*r* = −0.214, *p =* 0.058		
	Current methadone dosage	*r* = −0.167, *p =* 0.141		
	SOWS score	*r* = 0.114, *p =* 0.319		
	BDI score	*r* = −0.025, *p =* 0.825	*r* = 0.019, *p =* 0.872	*r* = 0.002, *p =* 0.982
	STAI-S score	*r* = 0.045, *p =* 0.695	*r* = −0.165, *p =* 0.162	*r* = −0.038, *p =* 0.638
	STAI-T score	*r* = 0.115, *p =* 0.313	*r* = −0.152, *p =* 0.200	*r* = 0.020, *p =* 0.806
	UPPS-P1 score: positive urgency	*r* = 0.059, *p =* 0.608	*r* = −0.026, *p =* 0.830	*r* = 0.032, *p =* 0.698
	UPPS-P2 score: negative urgency	*r* = 0.145, *p =* 0.202	*r* = −0.094, *p =* 0.431	*r* = 0.036, *p =* 0.663
	UPPS-P3 score: sensation seeking	*r* = −0.039, *p =* 0.732	*r* = 0.021, *p =* 0.857	*r* = 0.008, *p =* 0.922
	UPPS-P4 score: lack of premeditation	***r*** **=** **0.274**, ***p** **=*** **0.015**	*r* = −0.139, *p =* 0.242	*r* = 0.093, *p =* 0.254
	UPPS-P5 score: lack of perseverance	*r* = 0.193, *p =* 0.089	*r* = −0.073, *p =* 0.539	*r* = 0.069, *p = 0*.399
	SWM: number of errors	***r*** **=** **0.224**, ***p** **=*** **0.047**	*r* = −0.006, *p =* 0.961	*r* = 0.093, *p =* 0.252
	PAL: number of patterns reached	*r* = −0.045, *p =* 0.696	*r* = 0.012, *p =* 0.919	*r* = −0.038, *p =* 0.644
	PAL: number of errors	*r* = −0.073, *p =* 0.520	*r* = 0.063, *p =* 0.595	*r* = 0.010, *p =* 0.907
	SOC: number of moves (five-move trials)	*r* = 0.045, *p =* 0.697	*r* = 0.025, *p =* 0.837	*r* = 0.047, *p =* 0.564
	SOC: Trials solved in minimum moves	*r* = 0.006, *p =* 0.960	*r* = 0.149, *p =* 0.209	*r* = 0.059, *p =* 0.472

*A significant correlation (p < 0.05) is in bold. AUDIT, Alcohol Use Disorders Identification Test; BDI, Beck Depression Inventory; FTND, Fagerström Test for Nicotine Dependence; HC, healthy control; MMT, methadone maintenance therapy; PAL, Paired Associative Learning; SOC, Stockings of Cambridge; SOWS, Subjective Opiate Withdrawal Scale; STAI-S, State-Trait Anxiety Inventory-state; STAI-T, State-Trait Anxiety Inventory-trait; SWM, Spatial Working Memory*.

**Table 5:** AB scores in patient subgroups based on MMT duration and lack of premeditation subscale of UPPS-P.

	**AB scores—negative (ms)**				
**MMT duration**	***n***	**M (SD)**	**Range**	**Kruskal-Wallis statistic**	***P*-value**
≤1 year	6	14.7 (21.9)	−3.3-56.3	6.475	**0.039**
1-10 years	44	6.0 (27.3)	−37.5-122.4		
≥10 years	29	−6.2 (17.5)	−47.3-29.0		
**UPPS-P4: Lack of premeditation**					
<20	28	−2.6 (30.5)	−47.3-122.4	6.754	**0.034**
20-29	45	4.6 (20.3)	−37.5-93.2		
≥30	6	6.4 (21.6)	−29.3-29.0		

*A significant effect (p < 0.05) is in bold. AB, attentional bias; HC, healthy control; M, mean; MMT, methadone maintenance therapy; SD, standard deviation*.

## Discussion

In this study, we examine attentional bias to drug-related pictures and negative-affective pictures in opioid-dependent patients under chronic MMT. Moreover, its association with clinical, neuropsychological, and cognitive characteristics is investigated. Results indicate that tobacco smoking, dependence on nicotine, depression, anxiety, and impulsivity are all significantly more prevalent among the patients than the HCs. There are no significant overall patient-control group differences observed in the attentional bias to drug cues or negative-affective cues. In the patient group, however, the drug-related AB scores are associated with individual differences in impulsivity, as measured by the Lack of Premeditation UPPS-P subscale. The patients with relatively higher scores on the Lack of Premeditation obtain relatively higher negative-affective AB scores. A negative correlation is noted between the AB scores to negative-affective cues and the duration of MMT, indicating MMT may gradually reduce the subconscious reaction to the negative-affective cue. These results demonstrate normalized attentional bias in opioid-dependent patients on chronic MMT, suggesting chronic MMT may alleviate the abnormality of attentional bias in opioid-dependent patients. The results also highlight that the attentional avoidance of negative-affective cues is modulated by the duration of methadone treatment and the impulsivity level in the patients.

### Normalized Attentional Bias in Opioid-Dependent Patients Under Chronic MMT

Drug cue-induced cravings or negative affective feelings may induce drug use and relapse. The patients allocate more attention to drug-related information in the environment, which may result in partial or full relapse back to addictive behavior (1). Previous studies have indicated that drug-related attentional bias is associated with the severity of the addiction (2, 3), drug craving (4), and relapse to drug use after a period of abstinence (5). Attention control training may reduce attentional bias to drug cues and facilitate abstinence (7). Attentional bias to drug cues has been found in heroin addicts (3, 8), which may be mediated by enduring, perhaps permanent, changes within the brain's reward system (9). Moreover, substance use disorder patients show a higher level of depression, anxiety, and impulsivity (11, 12). Acute induction of negative affect increases heroin seeking in the addicts, particularly in those who report greater subjective reactivity to negative triggers (15), suggesting addicts whose mood is more negatively influenced by external stimuli are at higher risk for drug use.

Methadone maintenance is an effective replacement therapy to treat heroin addiction, which significantly reduces the relapse rate (16). The negative feelings caused by withdrawal symptoms and cravings for opioids can be reduced by methadone (17). Nevertheless, the studies investigating whether patients with opioid use disorder on MMT show abnormal reactivity to drug cues and negative-affective cues are limited. A review of the existing literature on attentional bias to drug addiction indicates less clear-cut and inconsistent results, which could result from methodological differences between the studies, or from differences in patient samples examined (19). Notably, the sample sizes of studies of opioid-addicted patients under MMT are relatively small (around 20), and the duration of MMT is relatively short or not specified (20, 21). Moreover, there is a lack of investigation on the relationship between attentional bias and demographic, therapeutic, and neuropsychological factors in the patients, for instance, anxiety and impulsivity, which are known to be associated with substance use and addiction (22–25).

In the present study, we employ a dot-probe detection task to assess drug-related and negative-affective-related attentional bias in opioid-addicted patients under chronic MMT, compared with matched healthy control participants (HCs). Compared with age, sex, and years of education-matched healthy controls, the opioid-dependent patients with chronic MMT show no significant difference in drug-cue or negative-affective-related attentional bias task (dot-probe task), suggesting chronic MMT may alleviate the abnormality of attentional bias in the opioid-dependent patients.

### Modulation of Therapeutic and Neuropsychological Factors on Attentional Bias

Although the patients show no significant difference in AB scores from HCs, the attentional bias to negative affective cues shows to be modulated by the duration of MMT therapy. Acute induction of negative affect increases heroin seeking in the addicts, particularly in those who report greater subjective reactivity to negative triggers (15), suggesting addicts whose mood is more negatively influenced by external stimuli are at higher risk for drug use. Our result indicates that attentional bias avoiding negative-affective pictures negatively correlates with the duration of MMT, with the patients receiving longer methadone treatment showing less attentional avoidance to negative-affective cues. Thus, this result may indicate that the specific subjective-pharmacological effects (distress blunting) of methadone have influenced response to negative-affective cues and suggest that MMT may gradually reduce the subconscious reaction to the negative-affective cue. Long-term MMT treatment may alleviate the impact of negative emotion on patients' attention.

Moreover, we observed that relatively higher negative-affect-induced AB scores are associated with relatively higher impulsivity subscale scores, namely Lack of Premeditation. In the conceptualization of impulsive personality provided by the UPPS-P (32), individuals who score high on the Lack of Premeditation subscale are characterized by a tendency to act without thinking. Attentional bias is thought to operate in a rapid and automatic manner, conceivably evoking immediate behavioral responses, especially in individuals who already have a tendency to react before the eliciting stimulus has been well-evaluated and in those individuals whose attention is easily captured by salient but incompatible with good performance stimulus events. Thus, the observed positive correlations suggest that patients with higher impulsivity are more likely to be affected by negative-affective cues, manifested as increased attentional avoidance. This result indicates that certain aspect of impulsivity is associated with increased tendencies to avoid negative, aversive stimuli.

### Limitations

The findings of the present study confirm and extend prior work but need to be interpreted with caution due to several limitations. First, the patients were not assessed before they entered MMT, making it impossible to draw any firm conclusions about the effects of MMT. For example, the patients show drug-related AB scores that are similar to those observed in the HCs. Although this finding may indicate that MMT effectively normalizes or reduces the attentional bias to drug cues in the patients, it could also be due to that the patients examined in the present study did not acquire an enduring drug-related attentional bias during their drug use history. Second, the study may have suffered from a lack of statistical power to detect certain effects in the data due to the relatively small sample sizes examined, even though the sample size is larger than the previous studies. Finally, we investigate quite a few effects and correlations but do not correct for inflated false-positive error rates from multiple comparisons. Thus, the present findings need to be replicated in future studies with larger sample size and with a patient population before the MMT.

## Conclusions

The results of the present study indicate that the patients under chronic methadone maintenance therapy show normalized attentional bias to drug and negative-affective cues, confirming the involuntary attention of the patients is not abnormally captured by external drug or negative-affective clues. Our findings also highlight that the attentional avoidance of negative-affective cues is modulated by the duration of methadone treatment and the impulsivity level in the patients. The present study suggests that methadone therapy can reduce the influence of illicit opioids on patients at the subconscious level. The related modulatory factors of attentional bias in the patients are also identified.

## Data Availability Statement

The datasets generated and analyzed in the present study are not publicly available because of data privacy regulations of patient data but are available from the corresponding authors upon reasonable request.

## Ethics Statement

The studies involving human participants were reviewed and approved by the Ethics Committees of Ruijin Hospital, Shanghai Jiao Tong University School of Medicine. The patients/participants provided their written informed consent to participate in this study.

## Author Contributions

VV, KW, CW, and CZ were responsible for the study concept and design. KW programmed the task. JL contributed to the acquisition of questionnaires and behavioral data. NZ assisted with data analysis and interpretation of findings. WL and JH drafted the manuscript. CW and CZ provided critical revision of the manuscript for important intellectual content. All authors critically reviewed the content and approved the final version for publication.

## Funding

CZ was supported by the fellowship of the Shanghai Research Center for Brain Science and Brain-Inspired Intelligence and Shanghai Clinical Research Center for Mental Health (19MC1911100). VV was supported by Medical Research Council Senior Clinical Fellowship (MR/P008747/1).

## Conflict of Interest

The authors declare that the research was conducted in the absence of any commercial or financial relationships that could be construed as a potential conflict of interest. The reviewer JD declared a shared affiliation, with no collaboration, with the authors JH and CZ at the time of the review.

## Publisher's Note

All claims expressed in this article are solely those of the authors and do not necessarily represent those of their affiliated organizations, or those of the publisher, the editors and the reviewers. Any product that may be evaluated in this article, or claim that may be made by its manufacturer, is not guaranteed or endorsed by the publisher.
